# Smoking-related changes in DNA methylation and gene expression are associated with cardio-metabolic traits

**DOI:** 10.1186/s13148-020-00951-0

**Published:** 2020-10-22

**Authors:** Silvana C. E. Maas, Michelle M. J. Mens, Brigitte Kühnel, Joyce B. J. van Meurs, André G. Uitterlinden, Annette Peters, Holger Prokisch, Christian Herder, Harald Grallert, Sonja Kunze, Melanie Waldenberger, Maryam Kavousi, Manfred Kayser, Mohsen Ghanbari

**Affiliations:** 1 Department of Epidemiology, Erasmus MC University Medical Center Rotterdam, 3015 GB Rotterdam, The Netherlands; 2 Department of Genetic Identification, Erasmus MC University Medical Center Rotterdam, 3015 GD Rotterdam, The Netherlands; 3 Research Unit of Molecular Epidemiology, Helmholtz Zentrum München, German Research Center for Environmental Health, 85764 Neuherberg, Germany; 4 Institute of Epidemiology, Helmholtz Zentrum München, German Research Center for Environmental Health, 85764 Neuherberg, Germany; 5 Department Internal Medicine, Erasmus MC University Medical Center Rotterdam, 3015 GD Rotterdam, the Netherlands; 6 German Center for Cardiovascular Research (DZHK), Partner Site Munich Heart Alliance, 80802 Munich, Germany; 7 Institute for Medical Informatics, Biometrics and Epidemiology, Ludwig-Maximilians-Universität (LMU) Munich, 80333 Munich, Germany; 8 Institute of Neurogenomics, Helmholtz-Zentrum München-German Research Center for Environmental Health, 85764 Neuherberg, Germany; 9 Institute for Clinical Diabetology, German Diabetes Center, Leibniz Center for Diabetes Research at Heinrich Heine University Düsseldorf, 40225 Düsseldorf, Germany; 10 German Center for Diabetes Research (DZD), 85764 Munich-Neuherberg, Germany; 11 Division of Endocrinology and Diabetology, Medical Faculty, Heinrich Heine University, 40225 Düsseldorf, Germany

**Keywords:** Cardio-metabolic traits, Epigenetics, DNA methylation, Gene expression, Smoking

## Abstract

**Background:**

Tobacco smoking is a well-known modifiable risk factor for many chronic diseases, including cardiovascular disease (CVD). One of the proposed underlying mechanism linking smoking to disease is via epigenetic modifications, which could affect the expression of disease-associated genes. Here, we conducted a three-way association study to identify the relationship between smoking-related changes in DNA methylation and gene expression and their associations with cardio-metabolic traits.

**Results:**

We selected 2549 CpG sites and 443 gene expression probes associated with current versus never smokers, from the largest epigenome-wide association study and transcriptome-wide association study to date. We examined three-way associations, including CpG versus gene expression, cardio-metabolic trait versus CpG, and cardio-metabolic trait versus gene expression, in the Rotterdam study. Subsequently, we replicated our findings in The Cooperative Health Research in the Region of Augsburg (KORA) study. After correction for multiple testing, we identified both *cis*- and *trans*-expression quantitative trait methylation (eQTM) associations in blood. Specifically, we found 1224 smoking-related CpGs associated with at least one of the 443 gene expression probes, and 200 smoking-related gene expression probes to be associated with at least one of the 2549 CpGs. Out of these, 109 CpGs and 27 genes were associated with at least one cardio-metabolic trait in the Rotterdam Study. We were able to replicate the associations with cardio-metabolic traits of 26 CpGs and 19 genes in the KORA study. Furthermore, we identified a three-way association of triglycerides with two CpGs and two genes (*GZMA*; *CLDND1*), and BMI with six CpGs and two genes (*PID1*; *LRRN3*). Finally, our results revealed the mediation effect of cg03636183 (*F2RL3*), cg06096336 (*PSMD1*), cg13708645 (*KDM2B*), and cg17287155 (*AHRR*) within the association between smoking and *LRRN3* expression.

**Conclusions:**

Our study indicates that smoking-related changes in DNA methylation and gene expression are associated with cardio-metabolic risk factors. These findings may provide additional insights into the molecular mechanisms linking smoking to the development of CVD.

## Background

Tobacco smoking is a major modifiable risk factor for premature death and non-communicable diseases worldwide [1]. With almost 18 million deaths in 2017, cardiovascular diseases (CVD) account for the largest number of deaths of non-communicable diseases [2]. Smoking is also associated with cardio-metabolic traits, such as dyslipidemia, hypertension, insulin resistance, and obesity, which are major risk factors leading to CVD [3, 4]. Furthermore, persistent smoking has an excessive impact on DNA methylation [5–7] and gene expression [8–10], which their alterations are also linked to cardio-metabolic traits and risk of CVD [11–16].

Extensive studies have shown the independent association of smoking with DNA methylation, gene expression levels, and disease risk. In this context, smoking is associated with alteration in DNA methylation levels of several genes related to type 2 diabetes [17] and coronary artery disease [18]. Additionally, smoking-related CpGs have a strong association with all-cause and cardiovascular mortality [19]. Nevertheless, much less research has investigated smoking-related changes in DNA methylation and gene expression concurrently and in relation to health outcomes. A recent study identified a link between smoking-related DNA methylation and gene expression changes with metabolic health [20]. Their results indicate possible molecular pathways in which smoking affects disease development.

In this study, we hypothesized that smoking-related modifications in DNA methylation and gene expression are associated with each other and, additionally, with cardio-metabolic traits. Hence, we first determined three-way associations, including CpGs versus gene expression, cardio-metabolic traits versus CpGs, cardio-metabolic traits versus gene expression. To this end, we selected CpGs and gene expression probes associated with current versus never smokers using the largest published epigenome-wide association study (EWAS) [6] and transcriptome-wide association study (TWAS) [8] to date. Next, we used data from the Rotterdam Study to test the expression quantitative trait methylation (eQTM) association between the selected CpGs and gene expression probes. Subsequently, we tested the association for these CpGs and genes with different cardio-metabolic traits, including lipids, glycemic indices, blood pressure, and obesity-related traits. Moreover, we performed mediation analysis to test the mediating effect of; (1) DNA methylation in the association between smoking and cardio-metabolic traits, (2) gene expression in the association between smoking and cardio-metabolic traits, and (3) DNA methylation in the association between smoking and expression levels of smoking-related genes. To test the validity of our findings, we further replicated our results in an independent cohort, The Cooperative Health Research in the Region of Augsburg (KORA) study.

## Results

**Fig. 1:**
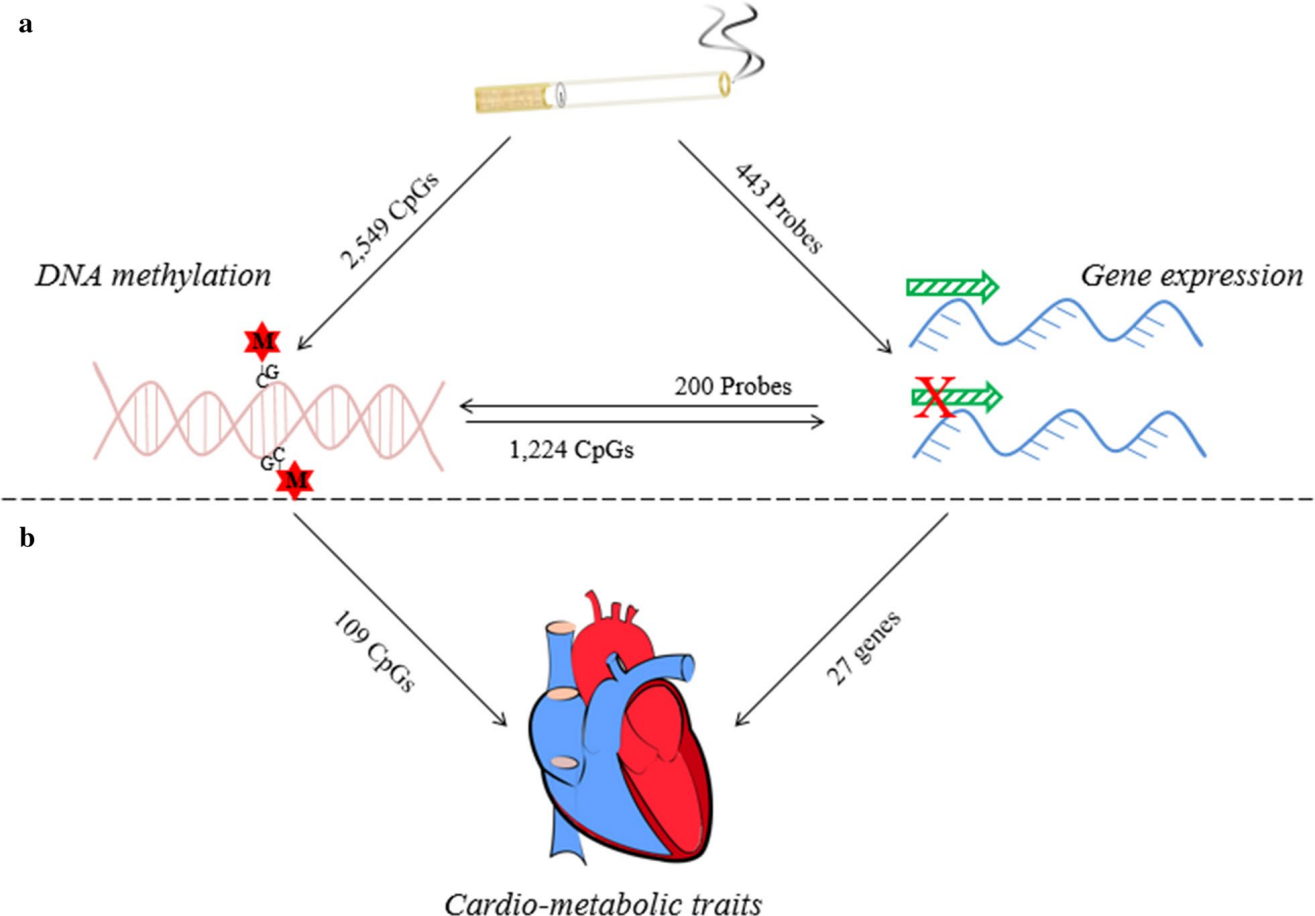
Schematic overview of the study design. In the current paper, previously identified CpGs by the largest available EWAS [6] and genes by the largest available TWAS [8] associated with current versus never smokers were used to test the link between smoking and cardio-metabolic traits. To this end, we first examined the association between smoking and alterations in gene expression (**a**). Second, we checked the association between the smoking-related CpGs and the smoking-related gene expression probes (**a**). Third, the smoking-related CpGs and gene expression probes that were in eQTM with each other were tested for their association with cardio-metabolic traits (**b**)

An overview of our study design is illustrated in Fig. 1. The discovery dataset consisted of 1412 participants with DNA methylation data from the two sub-cohorts of the Rotterdam study; RS-II and RS-III. Of these, 716 participants from RS-III had also gene expression data [21]. The replication dataset comprises 1727 participants with DNA methylation data, of whom 687 also had gene expression data, from the KORA study (F4) [22]. Both the discovery and replication cohorts consisted of both males and females (53.3%) and current, former and never smokers. In the current study, the former and never smokers are combined in the non-smoker category (83.6%). General characteristics of the study population are listed in Table 1.

**Table 1:** Population characteristics

	Discovery dataset		Replication dataset	
	Gene expression data set	DNA methylation data set	Gene expression data set	DNA methylation data set
*N*	716	1412	687	1727
Female	389 (54.3%)	791 (56.0%)	339 (49.3%)	882 (51.0%)
Age (years)	59.8 (± 8.1)	63.6 (± 8.1)	69.1 (± 4.4)	61.0 (± 8.8)
BMI (kg/m^2^)	27.6 (± 4.6)	27.7 (± 4.4)	28.9 (± 4.5)	28.1 (± 4.8)
WHR	0.9 (± 0.1)	0.9 (± 0.1)	0.9 (± 0.1)	0.9 (± 0.1)
Current smokers	193 (27.0%)	266 (18.0%)	53 (7.7%)	250 (14.5%)
Triglycerides (mmol/L)	1.5 (± 0.9)	1.5 (± 0.8)	1.5 (± 0.9)	1.5 (± 1.1)
HDL-cholesterol (mmol/L)	1.4 (± 0.4)	1.5 (± 0.4)	1.4 (± 0.4)	1.5 (± 0.4)
LDL-cholesterol (mmol/L)	3.9 (± 1.0)	3.8 (± 1.0)	3.7 (± 0.9)	3.6 (± 0.9)
Total cholesterol (mmol/L)	5.6 (± 1.1)	5.5 (± 1.0)	5.8 (± 1.0)	5.7 (± 1.0)
Lipid lowering medication (yes)	190 (26.5%)	404 (28.6%)	172 (25.0%)	283 (16.4%)
Systolic blood pressure (mm Hg)	134.2 (± 19.8)	139.5 (± 21.5)	128.7 (± 19.4)	124.8 (± 18.7)
Diastolic blood pressure (mm Hg)	82.8 (± 11.4)	83.6 (± 11.5)	74.7 (± 10.0)	76.1 (± 10.0)
Anti-hypertensive medication (yes)	215 (30.0%)	517 (36.6%)	383 (55.7%)	650 (37.6%)
Glucose (mmol/L)	5.6 (± 1.0)	5.6 (± 1.1)	NA	NA
Insulin (pmol/L)	96.0 (± 63.0)	89.3 (± 56.6)	88.2 (± 122.0)	81.3 (± 91.0)
Anti-diabetic medication (yes)	39 (5.4%)	95 (6.7%)	76 (11.1%)	134 (7.8%)

Values are presented as mean ± (SD) or *N* (%)

*BMI* body mass index, *WHR* waist to hip ratio, *HDL* high-density lipoproteins, *LDL* low-density lipoprotein

The participants included in the gene expression data are a subset of the total DNA methylation dataset

*NA* not applicable; the associations with glucose levels in model 2 from the discovery did not pass the significance threshold

### Correlation between smoking-related changes in DNA methylation and gene expression

We selected 2623 CpGs previously reported as being significantly (*P* < 1 × 10^–7^) differentially methylated between smokers and never smokers [6]. Of these, 2549 CpGs passed the quality control in the Rotterdam Study. Furthermore, we selected 502 gene expression probes that were differently expressed between smokers and never smokers (*FDR* < 0.05) and replicated in an independent dataset as part of the same study [8]. Of these, 443 gene expression probes passed quality control in the Rotterdam Study. Then, we investigated the eQTM associations to test the possible impact of smoking-related DNA methylation changes on the smoking-related genes, or vice versa. To this end, we computed the residuals for both the CpGs and gene expression probes. Then, we tested the association between all the smoking-related CpGs with all the smoking-related gene expression probes. Here, we investigated *cis*-eQTMs in which the CpG regulates transcription of a neighboring gene (≤ 250 Kb from each side of the transcription start site). Also, we studied the *trans*-eQTM association in which a CpG regulates distant genes located > 250 Kb of the transcription start site [23]. Notably, out of the 2549 smoking-related CpGs, 1224 were associated with at least one of the gene expression probes at the significance threshold of *P* < 4.4 × 10^–8^ (0.05/443 × 2549). Of the 443 tested gene expression probes, 200 probes were significantly associated with at least one of the 2549 CpGs, after correcting for multiple testing (Additional file 1: Table S1). The R code to generate the residuals for the CpGs and gene expression probes, and for the eQTM analysis are included in Additional file 2.

To examine the possible enrichment due to the smoking effect, we further tested if the number of significant eQTM associations is higher while using smoking-related CpGs and genes, compared to the number of eQTM associations while using randomly selected CpGs and genes. When testing the association between the 2549 smoking-related CpGs with 443 randomly selected gene expression probes, we found that only 325 CpGs are associated with at least one of these gene expression probes and 186 gene expression probes with at least one smoking-related CpG. Using the chi-square test of independence to compare the use of smoking-related gene expression probes versus randomly selected gene expression probes, we obtained for the CpGs (1224 vs 325, respectively) *P* < 1.0 × 10^–5^ and for the genes expression probes (200 vs 186, respectively) a *P* value of 0.38. Similarly, when testing the association between 2549 randomly selected CpGs with the 443 smoking-related gene expression probes, we found only 465 CpGs associated with at least one smoking-related gene expression probe, and 19 gene expression probes with at least one smoking-related CpG. Using the chi-square test of independence, comparing the use of smoking-related CpGs versus randomly selected CpGs, we found a significant difference (*P* < 1.0 × 10^–5^) for both the CpGs (1224 vs 465, respectively) and the gene expression probes (200 vs 19, respectively). These results indicate enrichment of smoking-related genes in smoking-related DNA methylation sites and vice versa.

The replication in the KORA study confirmed the association of 134 smoking-related CpGs with at least one gene expression probe and 50 smoking-related gene expression probes with at least one smoking-related CpG, after correcting for multiple testing, at the significance threshold of *P* < 2.04 × 10^–7^ (0.05/200 × 1224)*.*

### Association of smoking-related changes in DNA methylation and gene expression with cardio-metabolic traits

**Fig. 2:**
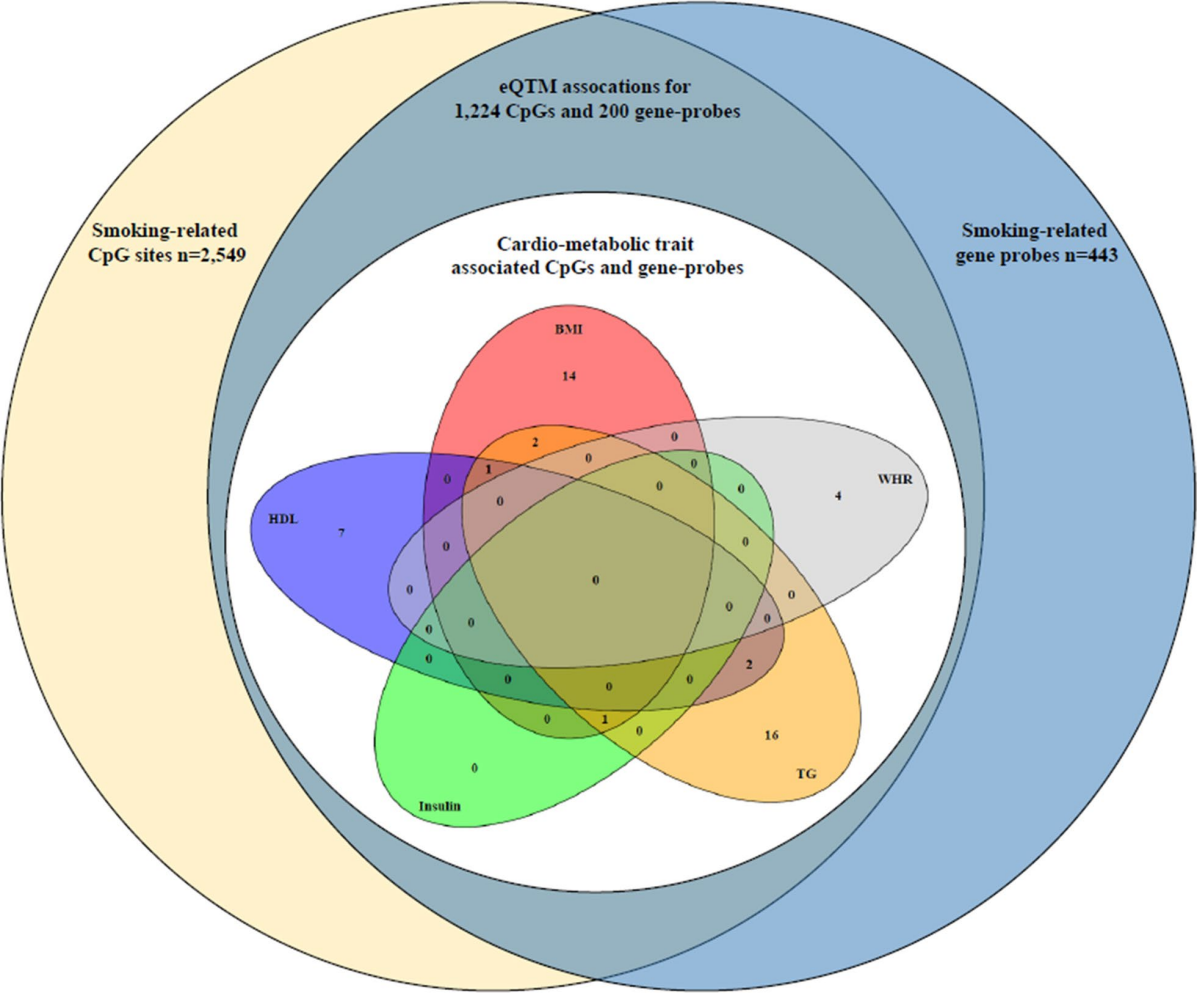
The overlap of smoking-related CpGs and genes in association with cardio-metabolic traits. In the current study, 2549 smoking-related CpGs and 443 smoking-related gene expression probes were included. Of these, 1224 CpGs and 200 gene expression probes showed eQTM association. We found the association for 26 CpGs and 19 genes (21 expression probes) with at least one cardio-metabolic traits, which surpassing the nominal significance (*P* < 0.05) in the KORA replication study

We tested the association of the 1224 CpGs and the 200 gene expression probes with cardio-metabolic traits, including high-density lipoprotein (HDL), low-density lipoprotein (LDL), triglycerides (TG) and serum cholesterol, fasting glucose and insulin levels, systolic blood pressure (SBP) and diastolic blood pressure (DBP), waist to hip ratio (WHR) and body mass index (BMI) in the Rotterdam Study. After adjusting for age, sex, blood cell count, and technical covariates (model 1), we found significant associations between 202 out of the 1224 smoking-related CpGs and any cardio-metabolic trait at *P* < 4.08 × 10^–5^ (0.05/1224) (*n* = 1412 participants) (Additional file 3: Table S2). Among these, we observed associations with HDL (126 CpGs), TG (84 CpGs), glucose (2 CpGs), insulin (10 CpGs), DBP (1 CpG), WHR (21 CpGs), and BMI (16 CpGs). After further adjustment for BMI and relevant medication in the model 2, associations with 109 CpGs remained significant, including HDL (58 CpGs), TG (35 CpGs), DBP (1 CpG), WHR (6 CpG), and BMI (16 CpG same as model 1) (Additional file 3: Table S3). The R code to test the association between cardio-metabolic traits and the CpGs are included in Additional file 4. We pursued replication in the KORA study for the CpGs reaching significance in the model 2 and found that 26 CpGs surpassed the nominal significance (*P* < 0.05, *n* = 1727 participants), including 8 CpGs for HDL, 8 CpGs for TG, 4 CpGs for WHR, and 7 CpGs for BMI (Table 2, Additional file 3: Table S3). The direction of associations with cardio-metabolic traits was consistent in all 26 replicated CpGs. Based on the stringent Bonferroni-adjusted *P* value threshold, the replication signals were significant at 2 CpGs for TG (*P* < 0.05/35 = 1.43 × 10^–3^), 3 CpGs for WHR (*P* < 0.05/6 = 8.33 × 10^–3^), and 4 CpGs with BMI (*P* < 0.05/16 = 3.13 × 10^–*3*^) (Table 2 and Fig. 2).

**Table 2:** CpG sites associated with cardio-metabolic traits in DNA methylation analysis

CpG	Chr:position^a^	Gene ID^b^	Trait	Model 1		Model 2		Replication	
				Effect	*P* value	Effect	P value	Effect	*P* value
cg04716530	16:30485684	*ITGAL*	HDL	0.01700	2.02E−07	0.01550	6.34E−06	0.00014	9.82E−04
cg07826859	7:45020086	*MYO1G*	HDL	0.01440	6.79E−06	0.01390	3.61E−05	0.00018	1.61E−03
cg26724967	16:3115223	*IL32*	HDL	0.01290	2.89E−06	0.01220	2.34E−05	0.00015	1.63E−03
cg16391678	16:30485597	*ITGAL*	HDL	0.01520	1.03E−06	0.01400	1.60E−05	0.00013	5.14E−03
cg16519923	16:30485810	*ITGAL*	HDL	0.01980	8.23E−08	0.01790	3.44E−06	0.00012	9.75E−03
cg10310310	7:157367150	*PTPRN2;MIR153-2*	HDL	0.01130	4.49E−06	0.01060	4.05E−05	0.00011	1.96E−02
cg24323726	3:111314186	*ZBED2;CD96*	HDL	0.01300	4.84E−07	0.01230	5.32E−06	0.00009	3.41E−02
cg07929642	16:89390685	*ANKRD11*	HDL	0.01650	1.87E−07	0.01550	3.13E−06	0.00009	4.25E−02
cg21566642	2:233284661	–	TG	− 0.01990	3.89E−05	− 0.02150	1.91E−05	− 0.01967	1.14E−05
cg04716530	16:30485684	*ITGAL*	TG	− 0.01370	1.23E−06	−0.01220	3.58E−05	−0.00380	5.34E−04
cg27409015	2:158114424	*GALNT5*	TG	0.01660	8.39E−07	0.01490	2.16E−05	0.00683	1.50E−03
cg06635952	2:70025869	*ANXA4*	TG	0.01300	9.06E−07	0.01280	3.36E−06	0.00502	5.95E−03
cg11095027	11:1297066	*TOLLIP*	TG	0.00996	2.72E−05	0.01040	2.64E−05	0.00375	7.82E−03
cg26219092	8:134388022	*–*	TG	0.01050	2.37E−06	0.00991	2.05E−05	0.00285	1.73E−02
cg10919522	14:74227441	*C14orf43*	TG	− 0.01410	7.90E−09	− 0.01260	7.97E−07	− 0.00491	1.91E−02
cg22635096	21:46550644	*ADARB1*	TG	0.01370	6.61E−08	0.01300	8.65E−07	0.00392	3.55E−02
cg00310412	15:74724918	*SEMA7A*	WHR	− 0.05000	3.96E−05	− 0.07150	1.95E−07	− 0.06952	4.54E−05
cg04424621	6:27101941	*HIST1H2BJ*	WHR	− 0.06490	1.06E−05	− 0.07360	1.03E−05	− 0.07191	5.73E−05
cg04583842	16:88103117	*BANP*	WHR	0.12600	5.29E−08	0.12500	1.56E−06	0.06802	3.71E−03
cg13755776	11:3602845	–	WHR	− 0.08530	1.03E−06	− 0.08200	3.33E−05	− 0.04521	3.71E−02
cg17287155	5:393347	*AHRR*	BMI	0.00117	8.69E−06	NA	NA	0.00053	3.11E−06
cg26361535	8:144576604	*ZC3H3*	BMI	0.00155	1.85E−07	NA	NA	0.00102	2.38E−05
cg06096336	2:231989800	*PSMD1;HTR2B*	BMI	0.00168	9.51E−07	NA	NA	0.00111	1.66E−04
cg13708645	12:121974305	*KDM2B*	BMI	0.00152	6.72E−07	NA	NA	0.00089	1.01E−03
cg25649826	17:20938740	*USP22*	BMI	0.00086	1.63E−05	NA	NA	0.00041	3.15E−03
cg24539517	10:121161258	*GRK5*	BMI	0.00149	2.95E−05	NA	NA	0.00078	4.33E-03
cg03636183	19:17000585	*F2RL3*	BMI	0.00160	3.04E−05	NA	NA	0.00063	3.40E−02

The table shows 26 CpGs that are associated to at least one cardio-metabolic trait and in eQTM with at least one smoking-related gene-expression probe

Only CpGs significantly associated in both models and nominally significant (*P* < 0.05) in the replication are presented in this table

*HDL* high-density lipoprotein, *TG* triglycerides, *WHR* waist to hip ratio, *BMI* body mass index, *NA* not applicable (because of adjusting for BMI)

Model 1: Adjusted for age, sex, cell count, and technical covariates. Model 2: Model 1 + BMI and relevant medication

We did not correct for additional covariates when testing the association for BMI

*P* value threshold for discovery *P* < 4.08 × 10^–5^ (0.05/1224)

*P* value threshold for replication: HDL, *P* < 8.62 × 10^–4^ (0.05/58); TG, *P* < 1.43 × 10^–3^ (0.05/35); WHR, *P* < 8.33 × 10^–3^ (0.05/6); BMI, *P* < 3.13 × 10^–3^ (0.05/16)

CpGs that are presented underlined passed the replication *P* value threshold in 1727 participants of the KORA study

^a^Genome coordinates provided by Illumina (GRCh37/hg19)

^b^According to the Illumina Infinium HumanMethylation450K annotation file

Furthermore, out of the 200 smoking-related gene expression probes 39 (35 genes) were significantly associated with at least one cardio-metabolic trait at *P* < 2.5 × 10^–4^ (0.05/200) in the Rotterdam Study (*n* = 716 participants) (Additional file 2: Table S4). In the Illumina HumanHT-12 Expression BeadChip array, some of the annotated genes have more than one probe. Therefore, we adjusted the analysis for the number of probes we tested and provided both the probe ID and annotated gene in the tables. Of the 39 probes, we found associations with HDL (15 genes), LDL (1 gene), TG (18 genes), cholesterol (1 gene), glucose (3 genes), insulin (13 genes), WHR (6 genes), and BMI (14 genes). After further adjustments in model 2, the associations of 29 probes (27 genes) remained significant, including HDL (5 genes), LDL (1 gene), TG (14 genes), cholesterol (1 gene), and insulin (2 genes), for the association with BMI nothing changed (14 genes) (Additional file 3: Table S5). The R code to test the association between cardio-metabolic traits and the gene expression probes is included in Additional file 5. Replication in the KORA study for the gene expression probes that reached significance in model 2 showed 21 probes (19 genes) that passed the nominal significance (*P* < 0.05, *n* = 687 participants). These include 2 genes for HDL, 13 genes for TG, 1 gene for insulin, and 10 genes for BMI (Table 3, Additional file 3: Table S5). The direction of the association between gene expression and cardio-metabolic traits was consistent for all these genes. Based on the stringent Bonferroni-adjusted P-value in which we adjusted for the number of probes, the replication signal was significant at 2 genes for HDL (*P* < 0.05/5 = 0.01), 11 genes for TG *(P* < 0.05/15 = 3.33 × 10^–3^), 1 gene for insulin (*P* < 0.05/2 = 0.025), and 4 genes for BMI (*P* < 0.05/16 = 3.13 × 10^–3^). Several of these genes were associated in model 2 with more than one cardio-metabolic trait and were replicated at least at the nominal significance (*P* < 0.05). For example, *KLRB1* (ILMN_2079655), *ITM2C* (ILMN_2366041), and *CD3D* (ILMN_2261416) were associated with both TG and BMI, and *OCIAD2* (ILMN_1700306) was associated with both HDL and TG, and *EFHD2* (ILMN_1761463) was associated with HDL, TG, and BMI (Table 3 and Fig. 2).

**Table 3:** Gene expression probes associated with cardio-metabolic traits

Probe ID	Gene ID^a^	Chr. ^a^	Trait	Model 1		Model 2		Replication	
				Effect	*P* value	Effect	*P* value	Effect	*P* value
ILMN_1700306	*OCIAD2*	4	HDL	− 0.4114	8.39E−07	− 0.3573	5.15E−05	− 0.0040	9.20E−05
ILMN_1761463	*EFHD2*	1	HDL	0.4668	1.60E−07	0.3576	0.00013	0.0022	1.38E−03
ILMN_2261416	*CD3D*	11	TG	0.9089	2.22E−15	0.8328	3.84E−12	0.3233	2.58E−15
ILMN_2079655	*KLRB1*	12	TG	1.1666	6.48E−14	1.0518	1.01E−10	0.3946	4.09E−15
ILMN_1779324	*GZMA*	5	TG	1.0562	1.99E−09	1.0033	6.01E−08	0.2734	2.06E−13
ILMN_1761463	*EFHD2*	1	TG	− 0.4238	2.59E−09	− 0.3434	3.54E−06	− 0.1298	9.99E−13
ILMN_1700306	*OCIAD2*	4	TG	0.3413	3.32E−07	0.2998	1.93E−05	0.1691	7.64E−10
ILMN_1808939	*RPS6*	9	TG	0.6145	5.58E−11	0.5687	7.54E− 09	0.3001	1.17E−09
ILMN_1812191	*C12orf57*	12	TG	0.4392	1.37E−05	0.3897	0.00023	0.1772	1.07E−07
ILMN_1776181	*BIRC3*	11	TG	0.6940	2.86E−10	0.6176	8.34E−08	0.1473	4.89E−07
ILMN_1813836	*DARS*	2	TG	0.2657	9.37E−07	0.2878	4.67E−07	0.0894	3.18E-06
ILMN_1669927	*ICOS*	2	TG	0.2859	6.88E-06	0.2692	5.75E-05	0.0925	2.95E−04
ILMN_2198878	*INPP4B*	4	TG	0.2950	2.99E−07	0.2853	2.53E−06	0.0668	5.93E−04
ILMN_2366041	*ITM2C*	2	TG	− 0.5648	6.81E−09	− 0.4159	3.35E−05	− 0.0818	6.33E−03
ILMN_1680453	*ITM2C*	2	TG	− 0.5880	5.50E−08	− 0.4295	0.000124	− 0.0818	7.05E−03
ILMN_2352563	*CLDND1*	3	TG	0.3877	4.61E−05	0.4003	6.52E−05	0.0656	3.53E−02
ILMN_2079655	*KLRB1*	12	Insulin	0.7694	7.01E−09	0.6393	5.21E−05	0.2120	5.59E−05
ILMN_1766657	*STOM*	9	BMI	0.0549	3.85E−07	NA	NA	0.0196	2.56E−08
ILMN_1671891	*PID1*	2	BMI	− 0.0425	7.23E−09	NA	NA	− 0.0137	3.27E−06
ILMN_2366041	*ITM2C*	2	BMI	− 0.0577	1.86E−09	NA	NA	− 0.0123	9.53E−05
ILMN_1773650	*LRRN3*	7	BMI	− 0.0669	3.77E−05	NA	NA	− 0.0162	1.70E−03
ILMN_1661599	*DDIT4*	10	BMI	− 0.0658	2.75E−07	NA	NA	− 0.0096	4.06E−03
ILMN_2048591	*LRRN3*	7	BMI	− 0.0604	1.46E−05	NA	NA	− 0.0086	6.95E−03
ILMN_2377669	*CD247*	1	BMI	− 0.0370	5.95E−05	NA	NA	− 0.0058	8.01E−03
ILMN_2109197	*EPB41L3*	18	BMI	− 0.0322	0.000112	NA	NA	− 0.0072	1.12E−02
ILMN_2261416	*CD3D*	11	BMI	0.0458	6.47E−05	NA	NA	0.0107	1.37E−02
ILMN_2079655	*KLRB1*	12	BMI	0.0669	1.63E−05	NA	NA	0.0122	2.29E–−02
ILMN_1761463	*EFHD2*	1	BMI	− 0.0339	1.46E−06	NA	NA	− 0.0038	4.68E−02

The table shows 21 probes annotated to 19 genes that are significantly associated with cardio-metabolic traits and in eQTM with at least one smoking-related CpG

Only probes significantly associated in both models and nominally significant (*P* < 0.05) in the replication are presented in this table

*HDL* high-density lipoprotein, *TG* triglycerides, *BMI* body mass index, *NA* not applicable (because of adjusting for BMI)

Model 1: Adjusted for age, sex, cell count, RNA quality score, and technical covariates. Model 2: Model 1 + BMI and relevant medication

We did not correct for additional covariates when testing the association for BMI

*P* value threshold *P* < 2.25 × 10^–4^ (0.05/200)

*P* value threshold for replication: HDL, *P* < 0.01, (0.05/5); TG, *P* < 3.33 × 10^–3^ (0.05/15); Insulin, *P* < 0.03 (0.05/2); BMI, *P* < 3.13 × 10^–3^ (0.05/16)

Genes that are presented underlined passed the replication *p* value threshold in 687 participants of the KORA study

^a^According to the by Illumina provided annotation file

Next, we explored whether there is an overlap in the results obtained with DNA methylation and gene expression data, which possibly explain the link between smoking and cardio-metabolic traits. Table 4 shows the overlap of the replicated association of cardio-metabolic traits with gene expression, which were both also associated with the smoking-related CpGs, indicating a three-way association (Fig. 1). Additional file 3: Table S6 displays the three-way association as obtained in our discovery dataset. For example, we found in the Rotterdam Study overlapping association of serum HDL levels with four CpGs (cg01305745, cg06177555, cg07990556, and cg16448702) and expression levels of three genes *(EFHD2*, *PRF1,* and *OSBPL5*). Likewise, we found the association of TG levels with 18 CpGs and six genes (*ICOS*, *GZMA*, *C12orf57*, *CD3D, CLDND1,* and *EFHD2*). Finally, we found BMI to be associated with 16 CpGs and five genes (*LRRN3*,* EFHD2*,* PID1*,* STOM*, and *CD3D*) (Additional file 3: Table S6). Of these, we were able to replicate the three-way association of TG with DNA methylation levels of cg04716530 and expression levels of *GZMA*, and DNA methylation levels of cg21566642 and expression levels of *CLDND1* in the KORA study. Furthermore, we found BMI to be associated with DNA methylation levels of 6 CpGs and expression of two genes (*LRRN3* and *PID1*) (Table 4).

**Table 4:** The DNA methylation sites associated with gene

Gene expression^a^			Trait	DNA methylation^b^			eQTM^c^	
ProbeID	Effect	*P* value		CpG	Effect	*P* value	Coeff	*P* value
ILMN_1779324 (GZMA)	1.0033	6.01E−08	TG	cg04716530	− 0.0122	3.58E−05	− 11.7641	6.91E−12
ILMN_2352563 (*CLDND1*)	0.4003	6.52E−05	TG	cg21566642	− 0.0215	1.91E−05	− 5.1957	3.54E−19
ILMN_1671891 (PID1)	− 0.0425	7.23E−09	BMI	cg03636183	0.0016	3.04E−05	− 3.9797	1.28E−11
ILMN_1773650 (LRRN3)	− 0.0669	3.77E−05	BMI	cg03636183	0.0016	3.04E−05	− 16.4622	3.46E−41
				cg06096336	0.0017	9.51E−07	− 15.0031	2.91E−24
				cg13708645	0.0015	6.72E−07	− 9.5025	2.31E−09
				cg17287155	0.0012	8.69E−06	− 26.2306	3.09E−54
				cg25649826	0.0009	1.63E−05	− 14.4989	3.43E−08
				cg26361535	0.0016	1.85E−07	− 12.549	2.30E - 11
ILMN_2048591 ( *LRRN3*)	− 0.0604	1.46E−05	BMI	cg03636183	0.0016	3.04E−05	− 14.4435	2.67E−43
				cg06096336	0.0017	9.51E−07	− 11.5428	1.40E−19
				cg13708645	0.0015	6.72E−07	− 8.2627	1.36E−09
				cg17287155	0.0012	8.69E−06	− 21.4408	1.19E−48

The table shows an overview of the overlap of the hits with nominal significant (*P* < 0.05) replication in KORA in all three association analyses, including the association between (1) DNA methylation and cardio-metabolic traits, (2) gene expression and cardio-metabolic traits, and (3) the eQTM results for the gene and CpG that are associated with the same cardio-metabolic trait

*P* value thresholds in the discovery for DNA methylation *P* < 4.08 × 10^–5^ (0.05/1224), gene expression *P* < 2.25 × 10^–4^ (0.05/200) and for eQTM *P* < 4.4 × 10^–8^ (0.05/443 × 2549)

*P* value thresholds in the replication for TG; gene expression *P* < 3.33 × 10^–3^ (0.05/15), DNA methylation *P* < 1.43 × 10^–3^ (0.05/35), and BMI; gene expression

*P* < 3.13 × 10^–3^ (0.05/16), DNA methylation *P* < 3.13 × 10^–3^ (0.05/16), and eQTM *P* < 2.04 × 10^–7^ (0.05/1224 × 200)

Results that are presented underlined passed the replication *P* value threshold in the KORA study

*TG* triglycerides, *BMI* body mass index

^a^Expression probe ~ cardio-metabolic trait + age, sex, cell count, RNA quality score, technical covariates, BMI and relevant medication

^b^CpGs ~ cardio-metabolic trait + age, sex, cell count, technical covariates, BMI and relevant medication

^c^Expression probe ~ CpGs + age, sex

In the three-way association (Table 4), we also identified CpGs associated with expression levels of genes far approximate from their annotated gene/loci. We did a lookup for the identified CpGs for eQTM association using data from the BIOS-BBMRI database (https://www.genenetwork.nl/biosqtlbrowser/). Here, we found *cis*-eQTMs between cg17287155 and expression of *EXOC3* and between cg03636183 and expression of *F2RL3*. In the Rotterdam Study, both *EXOC3* and *F2RL3* gene expression probes did not pass the QC. Hence, we could not test the influence of these genes in the identified eQTM associations in a three-way analysis.

### Mediation analysis for smoking-related CpGs and genes associated with cardio-metabolic traits

**Fig. 3:**
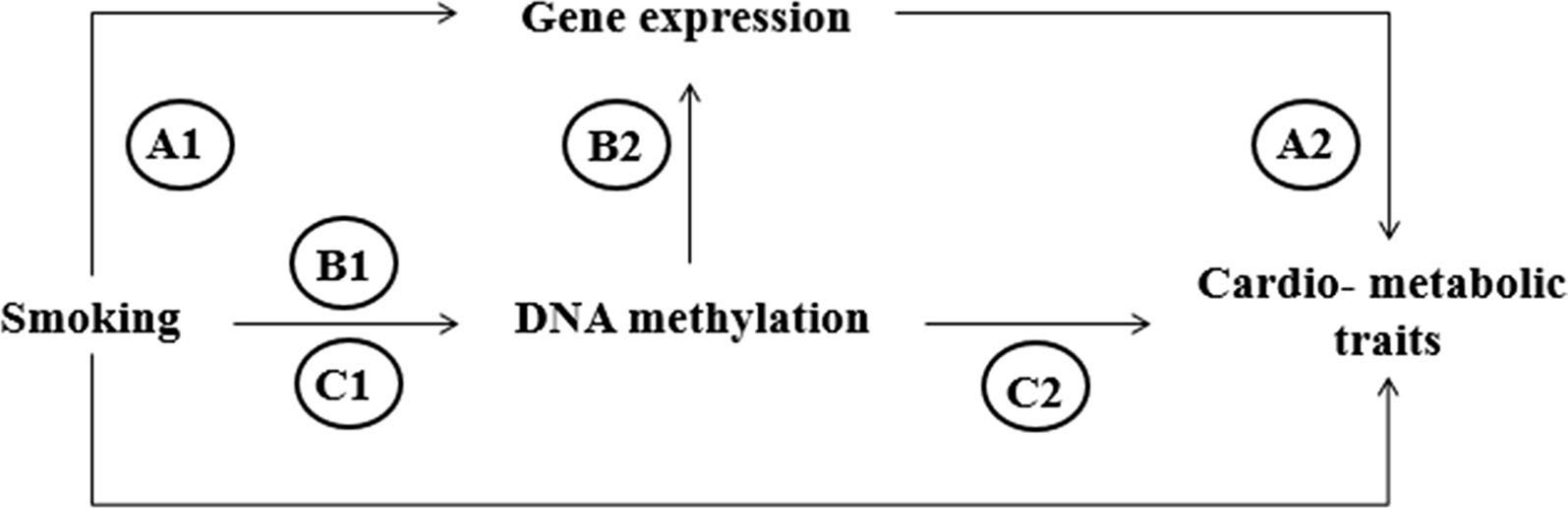
Schematic overview of the mediation analyses. We used mediation analysis to test the mediation effect of gene expression in the association between smoking and cardio-metabolic traits (A1 and A2). Furthermore, we tested the mediation effect of DNA methylation in the associations between smoking and gene expression (B1 and B2) and the mediation effect of DNA methylation in the association between smoking and cardio-metabolic traits (C1 and C2)

As shown in Fig. 3, we used mediation analysis to investigate the effect of DNA methylation and gene expression, independently, in the association between smoking and cardio-metabolic traits. Also, we tested the mediating effect of DNA methylation in the association between smoking and gene expression. In total, we conducted three different models; first, gene expression as a mediator in the observed association between smoking and cardio-metabolic traits (A1 and A2 in Fig. 3); second, DNA methylation as a mediator in the observed association between smoking and gene expression (B1 and B2 in Fig. 3); and third, DNA methylation as the mediator in the association between smoking and cardio-metabolic traits (C1 and C2 in Fig. 3). We conducted the average causal mediation effect (ACME), average direct effect (ADE), and the proportion mediated (Prop. med.), which are illustrated in Table 5 (and Additional file 3: Table S7). The ADE reflects the effect of smoking on the tested outcome that does not depend on the mediator. The R code for the mediation analyses is included in Additional file 6, and an example input file is provided in Additional file 7.

**Table 5:** Mediation effect of DNA methylation and gene expression in the association between smoking and cardio-metabolic traits

Mediator	Outcome	ACME (95% CI)	ADE (95% CI)	Total effect (95% CI)	Prob. Med. (95% CI)	*ρ* at which ACME is 0^a^
cg03636183 *(F2RL3)*	ILMN_1773650 *(LRRN3)*	0.6835 (0.4731/0.8869)	1.9603 (1.5832/2.3822)	2.6438 (2.2907/3.0048)	0.2585 (0.1767/0.3432)	− 0.3
cg06096336 *(PSMD1;HTR2B)*	ILMN_1773650 *(LRRN3)*	0.1237 (0.0263/0.2396)	2.5202 (2.1796/2.849)	2.6438 (2.2907/3.0048)	0.0468 (0.0102/0.0886)	− 0.4
cg13708645 *(KDM2B)*	ILMN_1773650 *(LRRN3)*	0.0768 (0.025/0.1408)	2.5671 (2.2153/2.9309)	2.6438 (2.2907/3.0048)	0.0290 (0.0092/0.0533)	− 0.1
cg17287155 *(AHRR)*	ILMN_1773650 *(LRRN3)*	0.6357 (0.4798/0.8094)	2.0081 (1.6771/2.333)	2.6438 (2.2907/3.0048)	0.2405 (0.1835/0.3036)	− 0.5
cg06096336 *(PSMD1;HTR2B)*	ILMN_2048591 *(LRRN3)*	0.0992 (0.0198/0.1915)	2.2838 (1.9578/2.5975)	2.3830 (2.0445/2.721)	0.0416 (0.0085/0.0779)	− 0.3
cg17287155 *(AHRR)*	ILMN_2048591 *(LRRN3)*	0.5004 (0.3828/0.6542)	1.8826 (1.5691/2.2123)	2.3830 (2.0445/2.721)	0.2100 (0.1603/0.2724)	− 0.4

The table shows the results of mediation analysis, in which current smoking is always used as exposure and is adjusted for age and sex

*ACME* average causal mediation effect, *ADE* average direct effect, *Prop. Med* proportion mediated

^a^ρ at which ACME is 0 indicates how sensitive our model is to the non-unmeasured confounding assumption

The mediation effect of the three-way associations as obtained in our discovery dataset (Additional file 3: Table S6) is provided in Additional file 3: Table S7. Out of the 124 mediation analysis conducted, there was significant mediation effect in 69 of them in the Rotterdam Study (Additional file 3: Table S7). Of these, we were able to replicate the mediating effect of cg01305745 (*VKORC1*) and cg16448702 (*INPP5D*) in the association between smoking and *PRF1* expression (ILMN_1740633). Also, we identified the mediating effect of cg16448702 (*INPP5D*) in the association between smoking and *OSBPL5* (ILMN_1802151). Furthermore, we replicated the mediation effect of 9 CpGs in the association between smoking and *LRRN3* expression (ILMN_1773650 and ILMN_2048591) (Additional file 3: Table S7). Finally, of the replicated three-way associations as shown in Table 4, we were able to replicate the mediation effect of cg03636183 (*F2RL3*), cg06096336 (*PSMD1; HTR2B*), cg13708645 (*KDM2B*), and cg17287155 (*AHRR*) in the association between smoking and *LRRN3* expression (Table 5). We conducted the ρ at which ACME is 0, to test the models' sensitivity. Here, we obtained *ρ*'s in the range between − 0.1 and − 0.5, and 0.1 and 0.4. A value of *ρ* close to 0 indicates that the assumption we made is sensitive to violations [24].

## Discussion

The associations of smoking, gene expression, and DNA methylation with cardio-metabolic traits have been studied independently and reviewed in great detail [11, 25–28]; however, the overlap between epigenetics and transcriptomics in the association between smoking and cardio-metabolic traits has been studied much less. This study investigated the relationship between previously identified smoking-related changes in DNA methylation [6] and gene expression [8], followed by their associations with cardio-metabolic traits within two population-based cohort studies. In this line, we first showed several significant *cis*- and *trans-*eQTM associations between smoking-related CpGs and gene expression probes. Furthermore, we replicated 26 smoking-related CpGs and 19 smoking-related genes (21 probes) associated with cardio-metabolic traits. Moreover, we showed three-way association of TG with two CpGs and two genes (*GZMA* and *CLDND1*), and BMI with six CpGs and two genes (*PID1* and *LRRN*). Finally, our study demonstrated a mediating effect of 4 CpGs (cg03636183, cg06096336, cg13708645, and cg17287155) in the association between smoking and the BMI-related gene *LRRN3*.

Our results showed a three-way association between TG with the decrease in DNA methylation levels of cg21566642 and the increase in expression levels of *CLDND1*. In this line, smoking was associated with an increase in the expression of *CLDND1* [8] and a decrease in cg21566642 DNA methylation levels [6]; and here, we showed the inverse relation between *CLDND1* expression and methylation levels at cg21566642. The expression of *CLDND1*, a tight junction protein, is shown to be highly increased in human Colon cancer samples and cell lines, and also positively correlated with tumor growth and disease progression [29]. The inverse association between DNA methylation levels at cg21566642 and smoking was previously shown in blood samples with cross-tissue replications in adipose tissue and skin tissue [20]. Additionally, cg21566642 is inversely associated with CVD risk [30], all-cause mortality [31], and with left ventricular mass (LVM) index in young adults [32]. LVM index is an important cardiac remodeling trait that is an intermediate phenotype for heart failure. In line with this, an increased LVM index is associated with high levels of TG [33, 34] and with an increased risk of depressed left ventricular ejection fraction, coronary heart disease, congestive heart failure, and stroke [35, 36].

In the three-way association for BMI, we found that smoking is associated with lower BMI, indicating that current smokers are less likely to be obese than never smoker, which has been reported in several previous studies as well [37–39]. Our results further showed that cg03636183 (*F2RL3*) was positively associated with BMI and negatively associated with the expression of *PID1* and *LRRN3*. Smoking was inversely associated with cg03636183 [6] and positively with *PID1* and *LRRN3* expression [8]. Here, we found an inverse relation between cg03636183 and expression levels of *PID1* and *LRRN3*. Due to the quality control implemented within the Rotterdam Study gene expression profiling data, gene expression data on *F2RL3* was not available. Therefore, we could not test if the association of cg03636183 with *PID1* and *LRRN3* expression levels was independent or via a downstream effect of *F2RL3* expression*.* Nonetheless, the inverse correlation between DNA methylation levels at cg03636183 and expression of *F2RL3* was previously shown [20]. This might indicate that the identified eQTM associations are, at least partly, via *F2RL3* expression*. F2RL3* encodes the protease-activated receptor-4 (PAR-4), a protein expressed in various tissues that introduce platelet activation, intimal hyperplasia, and inflammation [40]. Furthermore, the expression of *F2RL3* was associated with metabolic disease risk phenotypes, including a negative association with visceral fat mass and a positive association with total fat mass and android-to-gynoid fat ratio [20]. Additionally, the inverse association between DNA methylation levels at cg03636183 and smoking has been shown in blood samples with cross-tissue replications in adipose and skin tissues [20]. The inverse relation between DNA methylation levels at cg03636183 and TG [41], all-cause mortality [31], lung cancer incidence and mortality [42], as well as total mortality and cardiovascular mortality [43] was also previously identified. Also, a smoking-related decrease in cg03636183 methylation levels appears to increase serum levels of IL-18 [44]. IL-18 promotes the synthesis of IL-6, which stimulates the production of serum CRP [45, 46]. The increase in IL-18 and IL-6 leads to a higher risk ratio for CHD development [47]. Moreover, the increase in serum CRP concentrations results in increased risk ratios for CHD, ischemic stroke, vascular mortality, and non-vascular mortality [48].

Two of the CpGs, cg26361535 (*ZC3H3*) and cg25649826 (*USP22*), for which we found a three-way association with BMI and *LRRN3*, have been reported to be positively associated with BMI [49]. Both CpGs are cross-tissue replicated in adipose tissue and in isolated adipocytes for obese cases versus normal-weight controls. The association with cg26361535 was in the same direction and for cg25649826 in the opposite direction as obtained in our results [49]. Additionally, both CpGs were positively associated in blood with weight, WHR, glucose, insulin, TG, and CRP, and negatively with HDL. Furthermore, cg26361535 was positively associated with SBP and DBP [49] and all-cause mortality [31].

Finally, we identified a three-way association between BMI, an increase in methylation levels at cg17287155 (*AHRR*), and *LRRN3* expression. Smoking is negatively associated with DNA methylation levels at cg17287155 [6] and, as we replicated here, positively associated with BMI [50]. Notably, in the eQTM look-up we found a *cis*-eQTM for cg17287155 with the expression of *EXOC3,* instead of with its annotated gene (*AHRR*)*. AHRR* is a well-studied gene in relation to smoking [5] and is a key regulator of the Xenobiotic metabolism pathway responsible for detoxification of polyaromatic hydrocarbons (PAHs) in tobacco smoke [51, 52]. Nevertheless, *EXOC3* overexpression increases insulin-induced glucose uptake in adipocytes [53], indicating a possible link for *EXOC3* with CVD-related risk factors. Further research is needed to verify the eQTM-associations for cg17287155 with *EXOC3* and its impact on the eQTM-associations identified in the current study.

The identified associations and mediating effects in our study indicate a possible regulatory effect of DNA methylation on the expression levels of genes far from the neighboring methylation site, which so-called *trans*-regulatory effect of methylated CpG sites on gene expression [54]. So far, most previous studies have limited their research to the correlation between gene expression and DNA methylation at CpGs located in the nearby regions and in the gene body, or the *cis*-regulatory effect. In this line, a recent study has shown the *trans*-regulatory effect of DNA methylation in the associations with gene expression and chronic obstructive pulmonary disease [54]. Therefore, future research is needed with a broader methodological approach, including examining possible *trans*-regulatory effects to gain more insight into the epigenetic regulatory effects in disease studies.

This study has strengths as well as limitations that should be considered when interpreting the results. The main strengths of this study include the availability of DNA methylation data in a large sample of adults from the general population overlapping with transcriptomic and clinical data. Another strength is the use of the largest available EWAS [6] and TWAS [8] to date for selecting the CpGs and genes of interest associated with smoking. A limitation of the current study could be that data on smoking habits are retrieved from questionnaires, which might be underestimating actual smoking levels possibly leading to information bias [55–57]. This self-reporting bias can arise due to several reasons, such as recall bias in which a participant might not remember the true exposure or social desirability bias in which participants deliberately underestimate due to the socially stigmatized nature [57]. However, we expect the underestimation to be primarily quantitative and should not significantly impact the current versus non-smoker categorization we used in this study. Also, the questionnaires used for smoking data-collection did not include information regarding passive smoking, which is a risk factor for CVD [58]. As a result, we were not able to adjust for the passive-smoking effect in our analysis. As these participants are included in the non-smoker group, this might have underestimated the true effect.

Furthermore, due to the nature of the current study we have included the same participants in all mediation analyses and have used the mediator and exposure measurements on the same time-point; therefore, we cannot rule out reverse causality. Another limitation is that DNA methylation and gene expression levels were only measured at baseline; hence, we have no access to pre-measurement covariates. Consequently, we could not further adjust our models without risking the adjustment of a mediator, which could explain the *ρ* values close to 0 we obtained in a subset of our models in the sensitivity analysis. However, we did include additional adjustments (e.g., BMI and relevant medication) in the association analysis between cardio-metabolic traits with DNA methylation and gene expression, indicating the robustness of the identified three-way associations. Also, due to the stringent quality control in the Rotterdam Study, we were not able to test the impact of the *cis*-eQTM genes in the identified eQTMs. Finally, the use of whole-blood for the quantification of DNA methylation and transcriptomics associated with smoking and cardio-metabolic traits could be a limitation, since DNA methylation and gene expression are tissue-specific. Nonetheless, these data from other tissues are currently not available in the majority of population-based studies including the two participating cohorts in this study.

## Conclusions

In this study, we tested the association of smoking-related changes in DNA methylation and gene expression with cardio-metabolic traits. We found a three-way association of TG and BMI with CVD-relevant CpG sites and genes. Our results may provide further insight into the possible molecular cascades linking smoking to metabolic risk factors leading to CVD. Further research is warranted to conduct experimental research on the molecular mechanisms of the impact of smoking on cardiovascular disease and its risk factors through changes in DNA methylation and gene expression levels.

## Methods

### Study population

The discovery data set comprised a total of 1412 participants included in the Rotterdam Study; the design from the Rotterdam Study has been described elsewhere [21]. Briefly, in 1990 all residents of Ommoord, a district in Rotterdam, aged 55 years and older, were invited for participation (RS-I). In 2000, the cohort was extended with participants who had reached the age of 55 years or who had moved into the district (RS-II). An additional group was invited in 2006, from the age of 45 years and older (RS-III). Participants have been re-examined every 3–4 years. In the current study, we used data from the third visit from RS-II (RS-II-3) and the first and second visit of RS-III (RS-III-1 and RS-III-2). In total, DNA methylation measurements of 1412 participants from RS-III-1, RS-II-3, and RS-III-2 were included in our analysis. Additionally, gene expression data were available for 716 participants included in RS-III-1. Smoking information was collected via self-reported questionnaires; additional data collection details are described in Additional file 8.

The replication data comprised a total of 1717 participants included in The Cooperative Health Research in the Region of Augsburg (KORA) study. The KORA study is a series of independent population-based epidemiological surveys and follow-up studies of participants living in the region of Augsburg, Southern Germany. The KORA F4 study, a 7-year follow-up study of the KORA S4 survey (examined 1999–2001), was conducted between 2006 and 2008. The standardized examinations applied in the survey have been described in detail elsewhere [21]. A total of 3080 subjects with ages ranging from 32 to 81 years participated in the examination. In a random subgroup of 1802 KORA F4 subjects, the genome-wide DNA methylation patterns were analyzed as described in Additional file 3. Smoking information was collected via self-reported questionnaires; additional data collection details are described in Additional file 8.

### DNA methylation data

DNA methylation in the Rotterdam Study and KORA study was extracted from whole peripheral blood and DNA methylation measurements were obtained using the Illumina Infinium Human Methylation 450K BeadChip (Illumina Inc, San Diego, CA, USA). The DNA methylation pre-processing procedures are described in Additional file 3. The methylation proportion of a CpG site was reported as a methylation *β*-value in the range of 0 to 1. Genome coordinates provided by Illumina (GRCh37/hg19) were used to identify independent loci.

In the current study, CpGs of interest were selected using a recent EWAS [6] investigating the association between tobacco smoking and changes in DNA methylation values in the epigenome. In total, 2623 CpG sites were identified as being significantly (*P* < 1 × 10^–7^) differentially methylated between smokers and never smokers. In the Rotterdam Study, 2549 out of the 2623 CpGs passed the quality control and are included in this study (Additional file 3: Table S8).

### RNA expression data

In the Rotterdam Study, RNA was isolated from whole blood and gene expression profiling was performed using the IlluminaHumanHT-12v4 Expression Beadchips (Illumina, San Diego, CA, USA). The expression dataset is available at Gene Expression Omnibus (GEO) public repository under the accession GSE33828: 881 samples are available for analysis. In KORA F4, total RNA was extracted from whole blood and the Illumina Human HT-12 v3 Expression BeadChip (Illumina, San Diego, CA, USA) was used for gene expression profiling [59]. A more detailed description is implemented in Additional file 8.

In the current study, genes of interest were selected using a previous TWAS testing the association between gene expression and current versus never-smoking status [8]. In this TWAS, the meta-analysis was performed on all transcripts with matching gene Entrez IDs. Employing a significance threshold of FDR < 0.05, 886 significant gene Entrez IDs were identified, of which 387 replicated in an independent dataset. Employing the annotation file provided by the Illumina (HumanHT-12_V4), we found 502 gene expression probes to be annotated to these gene Entrez IDs out of which 443 were present in the Rotterdam Study and were included in the current study (Additional file 8: Table S9).

### Correlation between DNA methylation and gene expression

Since DNA methylation and gene expression may affect each other (i.e., eQTMs), we tested the association between 2549 CpGs and 443 gene expression probes linked to smoking in participants who had both methylation and gene expression data available in the Rotterdam Study (*N* = 716). We regressed out age, sex, blood cell counts (fixed effect), and technical covariates (random effect) on the normalized beta-values of the CpGs and separately on the mRNA expression levels using a linear mixed model analysis. The association between the residuals of DNA methylation (independent variable) and gene expression (dependent variable) was examined using a linear regression model. The robust Bonferroni-corrected *P* value threshold for a significant association was *P* < 4.4 × 10^–8^ (0.05/443 × 2549).

Additionally, we randomly selected 443 gene expression probes from the IlluminaHumanHT12v4 Expression Beadchips, and 2549 CpGs from the Illumina Human 450K array, that were available in the Rotterdam Study. Using the same methods mentioned above, we tested the association between the 2549 smoking-related CpGs with the 443 randomly selected gene expression probes, and the association between 2549 randomly selected CpGs with the 443 smoking-associated gene expression probes. The chi-square test of independence was used to test possible enrichment for the smoking effect.

### Association of DNA methylation and gene expression with cardio-metabolic traits

We studied the relationship of cardio-metabolic traits with (1) smoking-CpGs associated with at least one smoking-gene probe, and (2) smoking-gene probes associated with at least one smoking-CpG. We included the following cardio-metabolic-related phenotypes: HDL, LDL, TG, serum cholesterol, fasting glucose and insulin levels, SBP, DBP, WHR, and BMI.

First, we tested the association between the smoking-related CpGs (dependent variable) with the cardio-metabolic traits (exposure variable) using linear mixed effects models (LME4 package in R). The selected covariates in model 1 with fixed effects were age, sex, and cell counts for granulocytes, lymphocytes and monocytes. Array number and position number on array were added in the model as covariates with random effect to correct for batch effect. In model 2, we additionally adjusted for BMI and relevant medication, including for lipid exposures (lipid-lowering medication), for glycemic traits (glucose-lowering medication), for SBP and DBP (lipid-lowering medication and anti-hypertensives, diuretics, beta-blockers, calcium channel blockers, and RAAS modifying agents).

Second, we tested the association between gene expression (dependent variable) and the cardio-metabolic traits (exposure variable) using linear mixed-effects models (LME4 package in R), adjusting for age, sex, blood cell counts (granulocytes, lymphocytes, and monocytes), RNA quality score and batch effect. In model 2, we additionally adjusted for BMI and relevant medication (as described for DNA methylation).

Third, we combined our EWAS and TWAS results and showed the obtained three-way association; CpG versus gene expression; cardio-metabolic trait versus CpG; cardio-metabolic trait versus gene expression. For the CpG versus gene expression, we did a lookup for the identified CpGs to identify possible *cis-* eQTM associations using data from five Dutch biobanks (BIOS-BBMRI database) in a total of 3841 whole blood samples (https://www.genenetwork.nl/biosqtlbrowser/).

### Mediation analysis

CpGs and gene expression probes associated with each other and associated with the same cardio-metabolic trait were reviewed in three mediation analyses (Fig. 3); (1) the mediation of gene expression in the association between smoking status and the cardio-metabolic trait, (2) the mediation of DNA methylation in the association between smoking status and gene expression changes, and (3) the mediation of DNA methylation in the association between smoking status and the cardio-metabolic trait. In all three analyses, we included the same participants, current versus non-smokers as exposure and all models are corrected for age and sex. In the first analysis, we used the gene expression as potential mediator and the cardio-metabolic trait as outcome. In the second analysis, we used DNA methylation as possible mediator and the gene expression as outcome. In the third analysis, we used DNA methylation as possible mediator and the cardio-metabolic trait as outcome. We used the “mediate” function in the mediation package in R [60], using the bootstrap method including 1000 simulations and confidence intervals using the BCa method [61]. The proportion mediated describes the average magnitude of indirect association between smoking status and the gene expression or cardio-metabolic trait attributed through changes in DNA methylation or gene expression relative to the average total association, and it is calculated by dividing the average causal mediation effect by the average total effect [62]. Asymptotic 95% confidence intervals (CI) were obtained from nonparametric bootstrapping with 1000 iterations. These mediation analyses assumed no additional unmeasured confounding; however, if unobserved variables confound the models, the unmeasured confounding assumption is violated. Therefore, we used the sensitivity analysis included in the mediation package using the “medsens” function conducted by varying the values of *ρ* and determine the *ρ* at which ACME is 0 per model. Obtaining a value of *ρ* close to 0 indicates that the assumption is sensitive to violations, meaning that having a confounder with a higher correlation than the value of *ρ*, the assumption of no additional unmeasured confounding likely does not hold [24].

### Replication in the KORA study

The identified associations in the Rotterdam Study were replicated using the same models in the KORA study. The adjustment for blood cell counts (monocytes, granulocytes, and lymphocytes) was based on Houseman estimates rather than laboratory measurements [63]. Furthermore, principal components were used to adjust for technical covariates rather than plate number and position on array.

### Statistical analysis

All analyses were performed using the statistical package R. The eQTM analysis, and the associations of the cardio-metabolic traits with smoking-related CpGs and genes were conducted in R (version 3.2.0) under a Linux operating system, using the “LME4” package (version 1.1-16) and the “parallel” package (version 3.2.0). The mediation analyses were conducted in R studio Desktop (version 3.2.0) under Windows operating system using the “mediation” package (version 4.4.6.). Data collection and related statistical methods are provided in Additional file 8.

## Supplementary information

**Additional file 1.** In Additional file 1 (Table S1), we show the expression quantitative trait methylation (eQTM) results per gene expression probe.

**Additional file 2.** In Additional file 2, we provided the R code we used for obtaining the residuals of the smoking-related CpGs and genes. Also, we provided the R code used to test the eQTM associations.

**Additional file 3.** In Additional file 3, we included supplementary Tables S2-S9. In Tables S2-S5, we show the results for the associations between cardio-metabolic traits with DNA methylation model 1 (Table S2) and model 2 (Table S3), and gene expression model 1 (Table S4) and model 2 (Table S5). In Table S6, we show all the three-way associations obtained in the discovery. In Table S7, we show the mediation results for all the three-way associations obtained in the discovery, and the results obtained in the replication. In Table S8, we show the CpGs, and in Table S9, we show the gene expression probes that were included in the current study as markers of interest.

**Additional file 4.** In Additional file 4, we provided the R code we used for testing the association between the smoking-related CpGs and the cardio-metabolic traits.

**Additional file 5.** In Additional file 5, we provided the R code we used for testing the association between the smoking-related gene expression probes and the cardio-metabolic traits.

**Additional file 6.** In Additional file 6, we provided the R code we used for the mediation analysis.

**Additional file 7.** In Additional file 7, we provided the input file we used in the discovery phase for mediation analysis.

**Additional file 8** In Additional file 8, we included supplementary methods about data collection in the RS and KORA.

## Data Availability

The DNA methylation dataset supporting the conclusions of this article can be requested at https://www.epib.nl/research/ergo.htm or contact M. Arfan Ikram (m.a.ikram@erasmusmc.nl) for the Rotterdam Study data. The expression dataset supporting the conclusions of this article is available at GEO (Gene Expression Omnibus) public repository under the accession GSE33828: 881 samples are available for analysis from the Rotterdam Study. The informed consent given by KORA study participants do not cover data posting in public databases. However, data are available upon request from KORA Project Application Self-Service Tool (https://epi.helmholtz-muenchen.de/). Data requests can be submitted online and are subject to approval by the KORA Board.

## Abbreviations

ACME: Average causal mediation effect
ADE: Average direct effect
BMI: Body mass index
CPACOR: Incorporating control probe adjustment and reduction of global correlation
CVD: Cardiovascular disease
DBP: Diastolic blood pressure
eQTM: Expression quantitative trait methylation
EWAS: Epigenome-wide association study
HDL: High-density lipoprotein
KORA: Cooperative Health Research in the Region of Augsburg
LDL: Low-density lipoprotein
LVM: Left ventricular mass
Prop. med.: Proportion mediated
SBP: Systolic blood pressure
TG: Triglycerides
TWAS: Transcriptome-wide association study
WHR: Waist to hip ratio

## References

[1] WHO. WHO global report on mortality attributale to tobacco. 2012:392.

[2] Collaborators GCoD (2018). Global, regional, and national age-sex-specific mortality for 282 causes of death in 195 countries and territories, 1980–2017: a systematic analysis for the Global Burden of Disease Study 2017. Lancet.

[3] Sun K, Liu J, Ning G (2012). Active smoking and risk of metabolic syndrome: a meta-analysis of prospective studies. PLoS ONE.

[4] Mottillo S, Filion KB, Genest J, Joseph L, Pilote L, Poirier P (2010). The metabolic syndrome and cardiovascular risk a systematic review and meta-analysis. J Am Coll Cardiol.

[5] Kaur G, Begum R, Thota S, Batra S (2019). A systematic review of smoking-related epigenetic alterations. Arch Toxicol.

[6] Joehanes R, Just AC, Marioni RE, Pilling LC, Reynolds LM, Mandaviya PR (2016). Epigenetic signatures of cigarette smoking. Circ Cardiovasc Genet.

[7] Zeilinger S, Kühnel B, Klopp N, Baurecht H, Kleinschmidt A, Gieger C (2013). Tobacco smoking leads to extensive genome-wide changes in DNA methylation. PLoS ONE.

[8] Huan T, Joehanes R, Schurmann C, Schramm K, Pilling LC, Peters MJ (2016). A whole-blood transcriptome meta-analysis identifies gene expression signatures of cigarette smoking. Hum Mol Genet.

[9] Charlesworth JC, Curran JE, Johnson MP, Goring HH, Dyer TD, Diego VP (2010). Transcriptomic epidemiology of smoking: the effect of smoking on gene expression in lymphocytes. BMC Med Genom.

[10] Vink JM, Jansen R, Brooks A, Willemsen G, van Grootheest G, de Geus E (2017). Differential gene expression patterns between smokers and non-smokers: cause or consequence?. Addict Biol.

[11] Dhana K, Braun KVE, Nano J, Voortman T, Demerath EW, Guan W (2018). An epigenome-wide association study of obesity-related traits. Am J Epidemiol.

[12] Braun KVE, Dhana K, de Vries PS, Voortman T, van Meurs JBJ, Uitterlinden AG (2017). Epigenome-wide association study (EWAS) on lipids: the Rotterdam Study. Clin Epigenet.

[13] Richard MA, Huan T, Ligthart S, Gondalia R, Jhun MA, Brody JA (2017). DNA methylation analysis identifies loci for blood pressure regulation. Am J Hum Genet.

[14] Liu J, Carnero-Montoro E, van Dongen J, Lent S, Nedeljkovic I, Ligthart S (2019). An integrative cross-omics analysis of DNA methylation sites of glucose and insulin homeostasis. Nat Commun.

[15] Chen BH, Hivert MF, Peters MJ, Pilling LC, Hogan JD, Pham LM (2016). Peripheral blood transcriptomic signatures of fasting glucose and insulin concentrations. Diabetes.

[16] Huan T, Esko T, Peters MJ, Pilling LC, Schramm K, Schurmann C (2015). A meta-analysis of gene expression signatures of blood pressure and hypertension. PLoS Genet.

[17] Ligthart S, Steenaard RV, Peters MJ, van Meurs JB, Sijbrands EJ, Uitterlinden AG (2016). Tobacco smoking is associated with DNA methylation of diabetes susceptibility genes. Diabetologia.

[18] Steenaard RV, Ligthart S, Stolk L, Peters MJ, van Meurs JB, Uitterlinden AG (2015). Tobacco smoking is associated with methylation of genes related to coronary artery disease. Clin Epigenet.

[19] Zhang Y, Schöttker B, Florath I, Stock C, Butterbach K, Holleczek B (2016). Smoking-associated DNA methylation biomarkers and their predictive value for all-cause and cardiovascular mortality. Environ Health Perspect.

[20] Tsai PC, Glastonbury CA, Eliot MN, Bollepalli S, Yet I, Castillo-Fernandez JE (2018). Smoking induces coordinated DNA methylation and gene expression changes in adipose tissue with consequences for metabolic health. Clin Epigenet.

[21] Ikram MA, Brusselle G, Ghanbari M, Goedegebure A, Ikram MK, Kavousi M (2020). Objectives, design and main findings until 2020 from the Rotterdam Study. Eur J Epidemiol.

[22] Holle R, Happich M, Löwel H, Wichmann HE, Group MKS (2005). KORA—a research platform for population based health research. Gesundheitswesen.

[23] Bonder MJ, Luijk R, Zhernakova DV, Moed M, Deelen P, Vermaat M (2017). Disease variants alter transcription factor levels and methylation of their binding sites. Nat Genet.

[24] Muthén BO, Muthén LK, Asparouhov T (2017). Regression and mediation analysis using Mplus.

[25] Joehanes R, Ying S, Huan T, Johnson AD, Raghavachari N, Wang R (2013). Gene expression signatures of coronary heart disease. Arterioscler Thromb Vasc Biol.

[26] Huan T, Zhang B, Wang Z, Joehanes R, Zhu J, Johnson AD (2013). A systems biology framework identifies molecular underpinnings of coronary heart disease. Arterioscler Thromb Vasc Biol.

[27] Burns DM (2003). Epidemiology of smoking-induced cardiovascular disease. Prog Cardiovasc Dis.

[28] Muka T, Nano J, Voortman T, Braun KVE, Ligthart S, Stranges S (2016). The role of global and regional DNA methylation and histone modifications in glycemic traits and type 2 diabetes: a systematic review. Nutr Metab Cardiovasc Dis.

[29] Dhawan P, Singh AB, Deane NG, No Y, Shiou SR, Schmidt C (2005). Claudin-1 regulates cellular transformation and metastatic behavior in colon cancer. J Clin Invest.

[30] Fernandez-Sanles A, Sayols-Baixeras S, Curcio S, Subirana I, Marrugat J, Elosua R (2018). DNA methylation and age-independent cardiovascular risk, an epigenome-wide approach: the REGICOR Study (REgistre GIroni del COR). Arterioscler Thromb Vasc Biol.

[31] Svane AM, Soerensen M, Lund J, Tan Q, Jylhava J, Wang Y (2018). DNA methylation and all-cause mortality in middle-aged and elderly Danish twins. Genes (Basel).

[32] Sabogal C, Su S, Tingen M, Kapuku G, Wang X (2020). Cigarette smoking related DNA methylation in peripheral leukocytes and cardiovascular risk in young adults. Int J Cardiol.

[33] Jorgensen PG, Jensen MT, Biering-Sorensen T, Mogelvang R, Galatius S, Fritz-Hansen T (2016). Cholesterol remnants and triglycerides are associated with decreased myocardial function in patients with type 2 diabetes. Cardiovasc Diabetol.

[34] de las Fuentes L, Waggoner AD, Brown AL, Davila-Roman VG (2005). Plasma triglyceride level is an independent predictor of altered left ventricular relaxation. J Am Soc Echocardiogr.

[35] Drazner MH, Rame JE, Marino EK, Gottdiener JS, Kitzman DW, Gardin JM (2004). Increased left ventricular mass is a risk factor for the development of a depressed left ventricular ejection fraction within five years: the Cardiovascular Health Study. J Am Coll Cardiol.

[36] Gardin JM, McClelland R, Kitzman D, Lima JA, Bommer W, Klopfenstein HS (2001). M-mode echocardiographic predictors of six- to seven-year incidence of coronary heart disease, stroke, congestive heart failure, and mortality in an elderly cohort (the Cardiovascular Health Study). Am J Cardiol.

[37] Molarius A, Seidell JC, Kuulasmaa K, Dobson AJ, Sans S (1997). Smoking and relative body weight: an international perspective from the WHO MONICA Project. J Epidemiol Community Health.

[38] Shimokata H, Muller DC, Andres R (1989). Studies in the distribution of body fat. III. Effects of cigarette smoking. JAMA.

[39] Dare S, Mackay DF, Pell JP (2015). Relationship between smoking and obesity: a cross-sectional study of 499,504 middle-aged adults in the UK general population. PLoS ONE.

[40] Leger AJ, Covic L, Kuliopulos A (2006). Protease-activated receptors in cardiovascular diseases. Circulation.

[41] Dekkers KF, van Iterson M, Slieker RC, Moed MH, Bonder MJ, van Galen M (2016). Blood lipids influence DNA methylation in circulating cells. Genome Biol.

[42] Zhang Y, Schottker B, Ordonez-Mena J, Holleczek B, Yang R, Burwinkel B (2015). F2RL3 methylation, lung cancer incidence and mortality. Int J Cancer.

[43] Zhang Y, Yang R, Burwinkel B, Breitling LP, Holleczek B, Schottker B (2014). F2RL3 methylation in blood DNA is a strong predictor of mortality. Int J Epidemiol.

[44] Jhun MA, Smith JA, Ware EB, Kardia SLR, Mosley TH, Turner ST (2017). Modeling the causal role of DNA methylation in the association between cigarette smoking and inflammation in African Americans: a 2-Step Epigenetic Mendelian Randomization Study. Am J Epidemiol.

[45] Gerdes N, Sukhova GK, Libby P, Reynolds RS, Young JL, Schonbeck U (2002). Expression of interleukin (IL)-18 and functional IL-18 receptor on human vascular endothelial cells, smooth muscle cells, and macrophages: implications for atherogenesis. J Exp Med.

[46] Jones SA, Novick D, Horiuchi S, Yamamoto N, Szalai AJ, Fuller GM (1999). C-reactive protein: a physiological activator of interleukin 6 receptor shedding. J Exp Med.

[47] Kaptoge S, Seshasai SR, Gao P, Freitag DF, Butterworth AS, Borglykke A (2014). Inflammatory cytokines and risk of coronary heart disease: new prospective study and updated meta-analysis. Eur Heart J.

[48] Emerging Risk Factors C, Kaptoge S, Di Angelantonio E, Lowe G, Pepys MB, Thompson SG (2010). C-reactive protein concentration and risk of coronary heart disease, stroke, and mortality: an individual participant meta-analysis. Lancet.

[49] Wahl S, Drong A, Lehne B, Loh M, Scott WR, Kunze S (2017). Epigenome-wide association study of body mass index, and the adverse outcomes of adiposity. Nature.

[50] Aslibekyan S, Demerath EW, Mendelson M, Zhi D, Guan W, Liang L (2015). Epigenome-wide study identifies novel methylation loci associated with body mass index and waist circumference. Obesity (Silver Spring).

[51] Larigot L, Juricek L, Dairou J, Coumoul X (2018). AhR signaling pathways and regulatory functions. Biochim Open.

[52] Vu AT, Taylor KM, Holman MR, Ding YS, Hearn B, Watson CH (2015). Polycyclic aromatic hydrocarbons in the mainstream smoke of popular U.S. cigarettes. Chem Res Toxicol.

[53] Ewart MA, Clarke M, Kane S, Chamberlain LH, Gould GW (2005). Evidence for a role of the exocyst in insulin-stimulated Glut4 trafficking in 3T3-L1 adipocytes. J Biol Chem.

[54] Yoo S, Takikawa S, Geraghty P, Argmann C, Campbell J, Lin L (2015). Integrative analysis of DNA methylation and gene expression data identifies EPAS1 as a key regulator of COPD. PLoS Genet.

[55] Connor Gorber S, Schofield-Hurwitz S, Hardt J, Levasseur G, Tremblay M (2009). The accuracy of self-reported smoking: a systematic review of the relationship between self-reported and cotinine-assessed smoking status. Nicotine Tob Res.

[56] Tripepi G, Jager KJ, Dekker FW, Zoccali C (2010). Selection bias and information bias in clinical research. Nephron Clin Pract.

[57] Althubaiti A (2016). Information bias in health research: definition, pitfalls, and adjustment methods. J Multidiscip Healthc.

[58] Khoramdad M, Vahedian-Azimi A, Karimi L, Rahimi-Bashar F, Amini H, Sahebkar A (2020). Association between passive smoking and cardiovascular disease: a systematic review and meta-analysis. IUBMB Life.

[59] Schurmann C, Heim K, Schillert A, Blankenberg S, Carstensen M, Dörr M (2012). Analyzing illumina gene expression microarray data from different tissues: methodological aspects of data analysis in the metaxpress consortium. PLoS ONE.

[60] Tingley D, Yamamoto T, Hirose K, Keele L, Imai K (2014). Mediation: R package for causal mediation analysis. J Stat Softw..

[61] DiCiccio TJ, Efron B (1996). Bootstrap confidence intervals. Stat Sci.

[62] Imai K, Keele L, Tingley D (2010). A general approach to causal mediation analysis. Psychol Methods.

[63] Houseman EA, Accomando WP, Koestler DC, Christensen BC, Marsit CJ, Nelson HH (2012). DNA methylation arrays as surrogate measures of cell mixture distribution. BMC Bioinform.

