# Supplementary material for: Smoking-related changes in DNA methylation and gene expression are associated with cardio-metabolic traits
Source: Clin Epigenetics. 2020 Oct 22;12:157. doi: 10.1186/s13148-020-00951-0 (PMC7579899; doi:10.1186/s13148-020-00951-0)
Supplement: Supplementary file 8 — Additional file 8 In Additional file 8, we included supplementary methods about data collection in the RS and KORA. [file 13148_2020_951_MOESM8_ESM.docx]

**Supplementary Methods**

***The Rotterdam Study***
***Data collection***
In our analysis, we considered cardio-metabolic traits, including high-density-lipoprotein (HDL), low-density-lipoprotein (LDL), triglycerides, serum cholesterol, fasting glucose and insulin levels, systolic blood pressure (SBP), diastolic blood pressure (DBP), waist to hip ratio (WHR), and body mass index (BMI). During visits to the research center, participants were medically examined and blood samples were taken. Concentrations of HDL, triglycerides, and total cholesterol were measured using an automated enzymatic method. LDL was calculated using the Friedewald formula ((total cholesterol − HDL  − (triglycerides/5)) (1). Height and weight were measured during the center visit, and BMI was calculated (kg/m^2^). During home visit interviews, data on tobacco smoking, dietary intake, and medication use were collected. Data on tobacco smoking was acquired from questionnaires in which participants were asked about past and present cigarette, cigar and pipe smoking behavior and were categorized as never, former or current smokers. In the current study we combined the former and never smokers in the non-smokers category. Information regarding medicine use was derived from both structured home interviews and linkage to pharmacy records. In the current study we use for anti-hypertensive medication a combination of anti-hypertensives, diuretics, beta-blockers, calcium channel blockers, and RAAS modifying agents.

***Statistical analysis***Triglyceride levels, glucose, and insulin were log transformed using a natural log to obtain a normal distribution. The glucose and insulin levels of participants with non-fasting blood samples were set to missing values. To reduce the possible bias induced by missing values, we implemented multiple imputations using the "mice" package in R (version 3.3.0). Missing DNA methylation beta-values in the eQTM analysis were set to the mean beta-CpG value. None of the imputed variables had more than 35% missing data. Values were imputed using fully conditional specification (Markov chain Monte Carlo method) with a maximum iteration number of 10 (N= 25 imputations). Multiple imputation procedures were used to predict normally distributed variables using Bayesian linear regression, non-normally distributed variables using predictive mean matching and binary or categorical variables using logistic regression (2).
***DNA methylation data***

DNA was extracted from whole peripheral blood samples (stored in EDTA tubes) by standardized salting out methods. Per sample, 500ng extracted DNA was bisulfite treated using the Zymo EZ-96 DNA methylation kit (Zymo Research, Irvine, CA, USA) and hybridized to the Illumina Human 450K array (Illumina, San Diego, CA, USA) (3), according to manufacturer’s protocol. The CPACOR (incorporating Control Probe Adjustment and reduction of global CORrelation) workflow was used for the preparation and normalization of the obtained array data (4). The minfi package was used in for background correction (5). The samples in which technical problems were observed (e.g. during bisulfite conversion, hybridization, extension or specificity and sex mismatch) were removed. Additionally, probes with a detection P-value above background (≥ 1×10^-16^) in >5% of the samples were removed. Finally, only samples with a call rate > 95% were included. The intensity values were quantile normalized for each of the six probe type categories separately: type I unmethylated red/green, type I methylated red/green, and type II red/green. The percentage of methylation (β-value) of a given cytosine was calculated using these normalized intensities as the proportion of the methylated intensity value, as the sum of methylated+unmethylated+100 intensities. Genome coordinates provided by Illumina (GRCh37/hg19) were used to identify independent loci. Processing of the Rotterdam Study DNA methylation samples was performed at the Genetic Laboratory of Internal Medicine, Erasmus University Medical Centre, Rotterdam.

***RNA expression data***

Whole blood was collected (PAXGene Tubes; Becton Dickinson, Erembodegem, Belgium) and total RNA was isolated (PAXGene Blood RNA kits; Qiagen, Venlo, the Netherlands). RNA samples were processed according to the manufacturer’s instructions. The RNA samples were analysed using the Labchip GX (Caliper, Hopkinton, MA, USA) and samples with a RNA quality score >7 were amplified, labelled (TotalPrep RNA; Ambion, Austin, TX, USA) and hybridised to the IlluminaHumanHT12v4 Expression Beadchips (Illumina, San Diego, CA, USA). The RNA samples were processed at the Genetic Laboratory of Internal Medicine, Erasmus University Medical Centre, Rotterdam. Gene expression data was quantile-normalized to the median distribution and log2-transformed. Probe and sample means were centered to zero. Genes were considered significantly expressed when detection p values calculated by GenomeStudio were less than 0.05 in >10% of all discovery samples, which added to a total number of 21,238 probes. Quality control was carried out using the eQTL-mapping pipeline (https://github.com/molgenis/systemsgenetics/tree/master/eqtl-mapping-pipeline) (6). The expression dataset is available at GEO (Gene Expression Omnibus) public repository under the accession GSE33828: 881 samples are available for analysis.

***The Cooperative Health Research in the Region of Augsburg (KORA) study***

***Data collection***

For KORA we considered cardio-metabolic traits, including high-density-lipoprotein (HDL), low-density-lipoprotein (LDL), triglycerides, serum cholesterol, fasting insulin levels, systolic blood pressure (SBP), diastolic blood pressure (DBP), waist to hip ratio (WHR), and body mass index (BMI), smoking and intake of medication. Participants completed a questionnaire with details on health status and underwent standardized examinations with blood samples taken as described elsewhere (7).

***Statistical analysis***

Triglyceride levels, glucose and insulin were log transformed using a natural log to obtain a normal distribution. The glucose and insulin levels of participants with non-fasting blood samples were set to missing values.

***DNA methylation data***

The EZ-96 DNA Methylation Kit (Zymo Research) was used, according to the manufacturer’s protocol, to bisulfite-convert 1 µg genomic DNA with the alternative incubation conditions recommended when using the Illumina Infinium Methylation Assay (Illumina Inc., San Diego, CA, USA.). Genome-wide DNA methylation was assessed using the Illumina HumanMethylation450 BeadChip, following the Illumina Infinium HD Methylation protocol. Data normalization was done following the CPACOR pipeline (4). In summary, 65 single-nucleotide polymorphism markers were excluded and background correction was done using the minfi (5) R package. Probes were set to N/A if the detection p-value ≥0.01 or number of beads ≤3 and samples were excluded if the detection rate was ≤0.95. The signal intensities were quantile normalized into the six different probe-type categories defined by colour channel, probe-type and M/U subtype (Type-I M red, Type-I U red, Type-I M green, Type-I U green, Type-II red, Type-II green).

***RNA expression data***

Gene expression profiling was performed using the Illumina Human HT-12 v3 Expression BeadChip (8). Total RNA was extracted from whole blood collected and stored in PAXgene tubes (BD, Heidelberg, Germany) under fasting conditions using the PAXgene Blood miRNA Kit (Qiagen, Hilden, Germany). Purity and concentration of RNA were determined using a NanoDrop ND-1000 UV-Vis Spectrophotometer (Thermo Scientific, Hennigsdorf, Germany). 500 ng of RNA was reverse transcribed into cRNA and thereby biotin-UTP-labeled using the Illumina TotalPrep-96 RNA Amp Kit (Ambion, Darmstadt, Germany). A total of 3000 ng of labelled cRNA was hybridized to the Illumina Human HT-12 v3 Expression BeadChip, followed by washing steps as described in the Illumina protocol. GenomeStudio V 2010.1 Gene Expression Module was used to impute missing values and for quality control. Quantile normalization and L2T was performed in R (log transformation and quantile normalization (9) using the lumi:1.12.4 package (10) from the Bioconductor open source software (http://www.bioconductor.org). The blood cell proportions were calculated from DNA methylation data using the method of Houseman et al. (11).

**References**

1. Friedewald WT, Levy RI, Fredrickson DS. Estimation of the concentration of low-density lipoprotein cholesterol in plasma, without use of the preparative ultracentrifuge. Clin Chem. 1972;18(6):499-502.

2. Rubin DB. Multiple Imputation for Nonresponse in Surveys. Wiley, editor. Hoboken, New Jersey: John Wiley & Sons, Inc.; 1987.

3. Sandoval J, Heyn H, Moran S, Serra-Musach J, Pujana MA, Bibikova M, et al. Validation of a DNA methylation microarray for 450,000 CpG sites in the human genome. Epigenetics. 2011;6(6):692-702.

4. Lehne B, Drong AW, Loh M, Zhang W, Scott WR, Tan ST, et al. A coherent approach for analysis of the Illumina HumanMethylation450 BeadChip improves data quality and performance in epigenome-wide association studies. Genome Biol. 2015;16:37.

5. Aryee MJ, Jaffe AE, Corrada-Bravo H, Ladd-Acosta C, Feinberg AP, Hansen KD, et al. Minfi: a flexible and comprehensive Bioconductor package for the analysis of Infinium DNA methylation microarrays. Bioinformatics. 2014;30(10):1363-9.

6. Westra HJ, Peters MJ, Esko T, Yaghootkar H, Schurmann C, Kettunen J, et al. Systematic identification of trans eQTLs as putative drivers of known disease associations. Nat Genet. 2013;45(10):1238-43.

7. Holle R, Happich M, Lowel H, Wichmann HE, Group MKS. KORA--a research platform for population based health research. Gesundheitswesen. 2005;67 Suppl 1:S19-25.

8. Schurmann C, Heim K, Schillert A, Blankenberg S, Carstensen M, Dörr M, et al. Analyzing illumina gene expression microarray data from different tissues: methodological aspects of data analysis in the metaxpress consortium. PLoS One. 2012;7(12):e50938.

9. Bolstad BM, Irizarry RA, Astrand M, Speed TP. A comparison of normalization methods for high density oligonucleotide array data based on variance and bias. Bioinformatics. 2003;19(2):185-93.

10. Du P, Kibbe WA, Lin SM. lumi: a pipeline for processing Illumina microarray. Bioinformatics. 2008;24(13):1547-8.

11. Houseman EA, Accomando WP, Koestler DC, Christensen BC, Marsit CJ, Nelson HH, et al. DNA methylation arrays as surrogate measures of cell mixture distribution. BMC Bioinformatics. 2012;13:86.
